# Preditores de Readmissão Hospitalar até 30 Dias de CRM em Banco de Dados Multicêntrico: Estudo de Coorte Transversal

**DOI:** 10.36660/abc.20230768

**Published:** 2024-08-21

**Authors:** Rene Augusto Gonçalves e Silva, Gabrielle Barbosa Borgomoni, Fabiane Letícia de Freitas, Adnaldo da Silveira Maia, Cleóstones Farias do Vale, Eva da Silva Pereira, Leonardy Guilherme Ibrahim Silvestre, Luís Roberto Palma Dallan, Luiz Augusto Lisboa, Luís Alberto Oliveira Dallan, Fabio Biscegli Jatene, Omar Asdrúbal Vilca Mejia, grupo de estudos REPLICCAR

**Affiliations:** 1 Hospital João XXIII Santarém PA Brasil Hospital João XXIII, Santarém, PA – Brasil; 2 Universidade de São Paulo Instituto do Coração do Hospital das Clínicas Faculdade de Medicina São Paulo SP Brasil Instituto do Coração do Hospital das Clínicas da Faculdade de Medicina da Universidade de São Paulo, São Paulo, SP – Brasil; 3 Instituto Dante Pazzanese de Cardiologia São Paulo SP Brasil Instituto Dante Pazzanese de Cardiologia, São Paulo, SP – Brasil

**Keywords:** Hospitais, Readmissão do Paciente, Miocárdio

## Abstract

**Fundamento:**

A análise de indicadores como taxa de readmissão hospitalar é crucial para aprimorar a qualidade dos serviços e gestão em processos hospitalares.

**Objetivo:**

Identificar as variáveis correlacionadas a readmissão hospitalar até 30 dias após cirurgia de revascularização miocárdica (CRM).

**Métodos:**

Estudo de coorte transversal no banco de dados Registro Paulista de Cirurgia Cardiovascular II (REPLICCAR II)(N=3.392), de junho de 2017 a junho de 2019. Avaliaram-se retrospectivamente 150 pacientes para identificar os fatores correlacionados a readmissão hospitalar até 30 dias após-CRM via regressão logística univariada e multivariada. As análises foram realizadas no software R, com significância de 0,05 e intervalos de confiança de 95%.

**Resultados:**

Cento e cinquenta pacientes foram readmitidos até 30 dias após a alta hospitalar de CRM (150/3.392, 4,42%) principalmente por infecções (mediastinite, ferida operatória e sepse) totalizando 52 casos (52/150, 34,66%), outras causas foram: complicações cirúrgicas (14/150, 9,33%) e pneumonia (13/150, 8,66%). Os preditores de readmissão identificados foram: O modelo de regressão multivariada apontou intercepto (OR: 1,098, p<0,00001), apneia do sono (OR: 1,117, p=0,0165), arritmia cardíaca (OR: 1,040, p=0,0712) e uso de balão intra-aórtico (OR: 1,068, p=0,0021) como preditores do desfecho, com uma AUC de 0,70.

**Conclusão:**

4,42% dos pacientes foram readmitidos pós-CRM, principalmente por infecções. Fatores como apneia do sono (OR: 1,117, p=0,0165), arritmia cardíaca (OR: 1,040, p=0,0712) e uso de balão intra-aórtico (OR: 1,068, p=0,0021) foram preditores de readmissão, com uma discriminação de risco moderada (AUC: 0,70).

**Figure:**
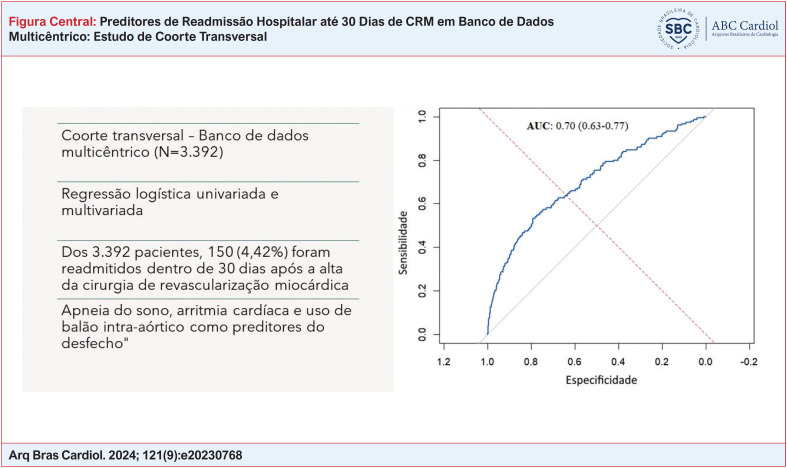


## Introdução

A avaliação do desempenho do sistema e dos processos hospitalares por meio de indicadores ajudam a gerenciar o hospital, contemplando não apenas a análise estrutural da instituição, mas também os processos e os resultados obtidos com o decorrer do tempo, e conforme as intervenções de qualidade contínua são aplicadas no serviço de saúde.^1,2^ Inicialmente, tais indicadores eram focados na mortalidade e complicações dos pacientes, entretanto, com os avanços dos conhecimentos do mundo moderno, variáveis como a readmissão hospitalar passaram a ser incluídas e avaliadas pelos gestores hospitalares.

A taxa de readmissão hospitalar, atualmente tem tido um importante papel no setor de qualidade dos hospitais, por se tratar de um indicador de desempenho no sistema de assistência, transparecendo a "análise de vida real" relacionada à qualidade do sistema, permitindo o monitoramento dos processos que culminam na reinternação do paciente e a definição de estratégias proativas para melhoria de resultados.^3^

A necessidade de retorno hospitalar pelo paciente recém-operado posterga a sua volta às atividades cotidianas, acarretando dificuldades no que tange à preservação da saúde e bem-estar geral do enfermo do ponto de vista psicológico e físico, expondo-o novamente^4^ ao ambiente hospitalar, potencialmente contaminado, aumentando a probabilidade de desfechos negativos,^5^ sobretudo na nova era COVID-19,^4^ além de representar impacto financeiro para o hospital.

A vista disto, levando em consideração que as readmissões são relativamente comuns após cirurgia de revascularização miocárdica (CRM), apresentando dados heterogêneos que oscilam entre 8,3 e 21,1%,^6-8^ mesmo com variação entre instituições, é importante identificar as razões da ocorrência deste evento, de forma de realizar ações proativas para redução destas taxas no futuro próximo, proporcionando melhor qualidade de atendimento e assistência ao paciente submetido a CRM e maior disponibilidade de leitos para novos pacientes, consequentemente aumentando a promoção de saúde para a população. Vale ressaltar que estudos abordando esta temática ainda são raros e dispersos na bibliografia brasileira, apontando significativa variedade de definições e uma lacuna no conhecimento científico da comunidade médica.

O objetivo deste estudo é identificar os fatores que pré-dispõem pacientes submetidos a CRM a readmissão hospitalar em até 30 dias após a alta hospitalar. Trata-se de um estudo de coorte transversal, sob ótica de uma análise retrospectiva do banco de dados multicêntrico do estado de São Paulo: Registro Paulista de Cirurgia Cardiovascular II (REPLICCAR II).

## Métodos

### Design do estudo

Estudo transversal do Registro Paulista de Cirurgia Cardiovascular II (REPLICCAR II),^9^ no qual a coleta de dados foi feita de forma prospectiva, observacional e multicêntrica de pacientes submetidos a CRM primária e isolada entre julho de 2017 e junho de 2019 em hospitais de referência no estado de São Paulo.

Os autores seguiram os critérios estabelecidos pelo *Strengthening the Reporting of Observational Studies in Epidemiology* (STROBE).^10^

Para esta análise, foram avaliados os pacientes que necessitaram ser reinternados em suas instituições de saúde até 30 dias após alta hospitalar de CRM, bem como os fatores que predispuseram a readmissão hospitalar após cirurgias de revascularização miocárdica.

**Figura 1:**
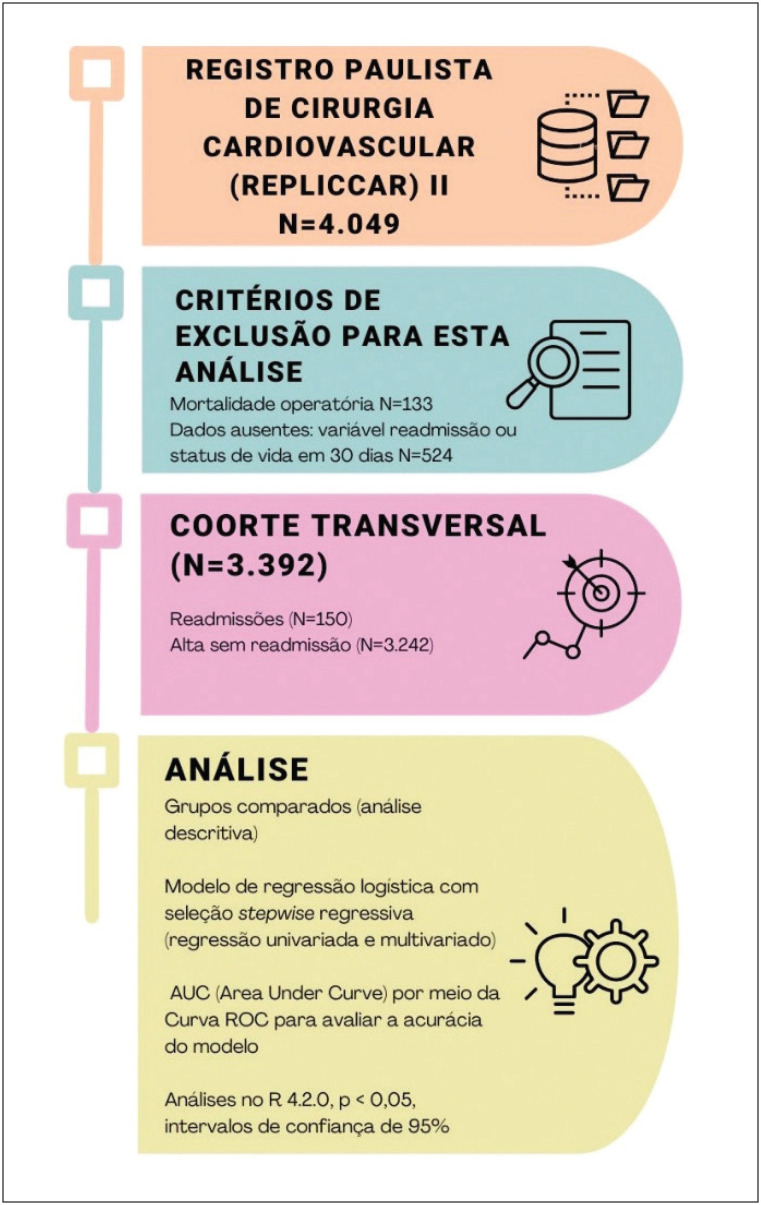
Fluxograma da pesquisa. Fonte: autoria própria.

O fluxograma de metodologias abordadas está disposto na figura 1, enquanto isso, a caracterização dos pacientes avaliados é representada no arquivo suplementar 1, comparando o perfil e a evolução da população que foi readmitida na instituição de saúde e a população que não precisou da reinternação hospitalar.

### Dados utilizados

Foram incluídos todos os pacientes submetidos a CRM, no período de recrutamento, com idade superior a 18 anos e com indicação cirúrgica isolada e primária (N=4.049). Todos os dados obtidos foram inseridos na plataforma *REDCap*, em uma área dedicada ao projeto REPLICCAR II, por profissionais de saúde treinados para desempenhar esta tarefa. Todas as definições das variáveis seguiram os critérios e definições estipulados pela versão 2.9 da STS *Adult Cardiac Surgery Database.*^11^ Auditorias de qualidade foram realizadas periodicamente com o intuito de verificar a exatidão, integridade e consistência dos dados.^12^

A variável morbidade foi um desfecho composto que incluiu os desfechos: acidente vascular cerebral, falha renal aguda, intubação prolongada, infeção profunda da ferida torácica e reoperação.

Critério de exclusão desta presente análise: mortalidade operatória (definida como a morte ocorrida após a alta hospitalar, mas antes do trigésimo dia de pós-operatório, assim como os óbitos ocorridos durante a internação em que a operação foi realizada, mesmo que após 30 dias depois do procedimento [N=133]) e dados ausentes (*missing*) na variável "readmissão’ ou status de vida até 30 dias após a alta hospitalar (N=524).

Para este estudo, foi realizada coorte entre os pacientes que necessitaram readmissão na instituição de saúde na qual foi submetido a CRM (N=150), e a partir de seus dados, as análises estatísticas de regressão foram realizadas para identificação das causas relacionadas à readmissão e os fatores associados ao evento. Os dados relacionados aos 3.242 pacientes que receberam alta sem readmissão nos 30 dias e permaneceram vivos até o final do acompanhamento foram comparados com o grupo readmitido.

### Ética e consentimento

Esta é uma subanálise do projeto REPLICCAR II, aprovado pela Comissão de Ética para Análises de Projetos de Pesquisa (CAPPesq) do Hospital das Clínicas da Universidade de São Paulo com o número 1.575, sob o número de registro SDC: 4506/17/006. O consentimento livre e esclarecido foi dispensado devido à metodologia do desenho de pesquisa aplicado ao projeto.

### Análise estatística

Na análise descritiva, as variáveis contínuas foram expressas em média ± desvio-padrão ou mediana e intervalo interquartil, conforme normalidade dos dados, em termos de medidas de resumo (média, mediana, desvio padrão e quartis), enquanto as variáveis categóricas foram expressas em termos de porcentagens. Como as variáveis contínuas não seguem distribuição normal (teste de Anderson-Darling), para a comparação de dois grupos foram utilizados os testes não paramétricos de Mann-Whitney e Brunner-Munzel, respectivamente, para as variáveis homogêneas e heterogêneas (teste de Bartlett). Para as variáveis categóricas, foi utilizado o teste Exato de Fisher ou o teste Qui-Quadrado.

Para encontrar associações entre as variáveis explicativas e desfecho binário foi utilizado o modelo de regressão logística. Para a construção de um modelo múltiplo mais parcimonioso (menos variáveis), foi utilizado o algoritmo de stepwise backward para selecionar as variáveis que melhor explicam o desfecho. Foi utilizado a AUC (Area Under Curve) por meio da Curva ROC para avaliar a acurácia do modelo.

Todas as análises foram realizadas com o software R versão 4.2.0. O nível de significância adotado nos testes foi de 0,05. Foram consideradas hipóteses bicaudais. Além disso, os intervalos de confiança construídos são de 95%.

## Resultados

Foi feita uma coorte transversal no banco de dados REPLICCAR II, originando 2 grupos: 1) Grupo de pacientes que foram readmitidos até 30 dias após a alta hospitalar (N=150, 4,42% da amostra total desta análise) e 2) pacientes que receberam alta e não necessitaram retornar ao hospital (N=3.242).

Na tabela suplementar 1 foram representadas as variáveis descritivas entre os 2 grupos analisados. Observamos que o grupo de pacientes que necessitou readmissão até 30 dias após a alta hospitalar apresentava maior quantidade de pacientes do sexo feminino (p<0,001), tendencia à anemia no período pré, intra e pós-operatório (p<0,001, p<0,001 e p=0,001), hemoglobina glicosilada mais elevada (p=0,013) e necessidade de transfusão sanguínea intraoperatória (p<0,001).

**Figura 2:**
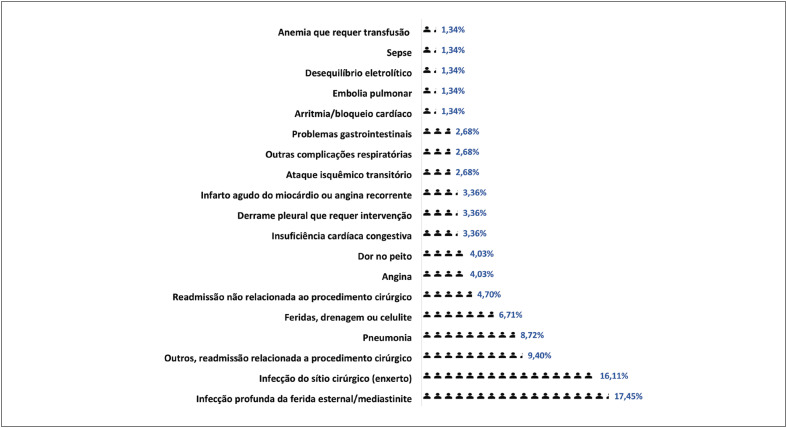
Causas de readmissão. Fonte: autoria própria.

Identificamos que dentre as razões que levaram os pacientes a retornar às instituições pertencentes ao estudo, os quadros infecciosos (mediastinite, ferida operatória e sepse) foram as mais expressivas (n=34,66%), como é representado na Figura 2 a seguir.

A análise de regressão foi feita a partir da variável de desfecho "readmissão hospitalar" no banco de dados REPLICCAR II. Com base nas variáveis significativas da regressão univariada (arquivo suplementar 2), o modelo multivariado foi construído (tabela 1). No modelo da regressão multivariada foram identificadas variáveis com impacto significativo sobre o desfecho em estudo. O intercepto mostrou-se significativo com um Odds Ratio (OR) de 1.098 (IC 95%: 1.065 - 1.131, p<0,00001), indicando um fator base para o desfecho quando todas as outras variáveis são mantidas constantes. A apneia do sono esteve associada a um aumento de 11.7% na chance do desfecho (OR: 1.117, IC 95%: 1.020 - 1.222, p=0,0165). Arritmia cardíaca apresentou um OR de 1.040 (IC 95%: 0.997 - 1.085, p=0,0712), embora essa associação não tenha alcançado significância estatística no nível de 0,05. A variável numérica do banco de dados em análise "hematócrito intraoperatório mais baixo registrado durante o período intraoperatório" foi associado a uma ligeira diminuição no risco do desfecho (OR: 0,994, IC 95%: 0,991 – 0,997, p=0,0004). O uso de balão intra-aórtico foi associado a um aumento de 6.8% na chance do desfecho (OR: 1.068, IC 95%: 1,024 – 1,113, p=0,0021).

**Tabela 1:** Regressão multivariada

Variável Explicativa	Coeficiente	Erro Padrão Coeficiente	OR	IC 95%	Valor de p
Intercepto	0,093	0,015	1,098	1,065-1,131	<0,00001
Arritmia Cardíaca	0,039	0,022	1,040	0,997-1,085	0,071212
Apneia do sono	0,110	0,0046	1,117	1,021-1,222	0,016523
Hemoglobina intraoperatória mais baixa registrada no período intraoperatório	-0,006	0,002	0,994	0,991-0,997	0,000399
Necessidade de Balão intra-aórtico	0,066	0,021	1,068	1,024-1,113	0,002103

IC: intervalo de confiança; Fonte: autoria própria.

**Figura 3:**
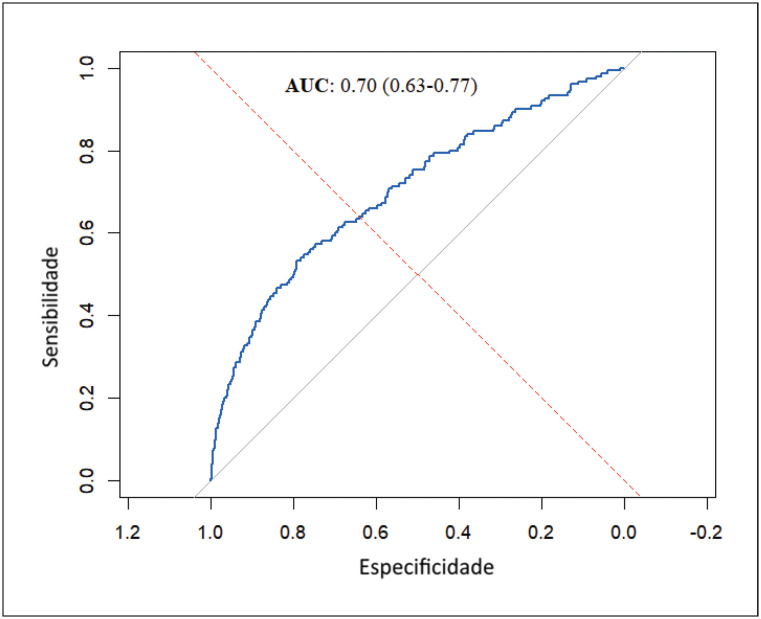
Curva ROC. Fonte: autoria própria.

Para testar a performance do modelo para predizer readmissões, foi utilizado o teste stepwise forward-backward. A curva ROC teve uma área sob a curva (AUC) de 0,70, indicando uma capacidade de discriminação moderada do modelo (IC 95%: 0,63 – 0,77). Esta AUC sugere que o modelo tem uma probabilidade de 70% de discriminar corretamente entre os desfechos positivo e negativo (Figura 3).

## Discussão

Nesta presente análise, foi observado que na coorte do banco de dados REPLICCAR II (N=3.392) houve 150 readmissões (4,42%). O que chama a atenção é que esta taxa é significativamente menor do que é observado na literatura científica atual, onde as taxas de readmissão variam entre 8,3 e 21,1%.^6-8^ Acreditamos que o fator explicativo deste fato, seja que o REPLICCAR II teve como premissa a melhoria de qualidade nos serviços de saúde associados ao banco, o que trouxe avanços positivos em diversos indicadores.^2,13^

Em 2012 Li et al.^14^ avaliaram 11.823 altas hospitalares após CRM na Califórnia e identificaram uma taxa de 13,2% de readmissões até 30 dias, correlacionando os seguintes fatores de risco: idade, gênero, renda familiar e código postal compatível a regiões mais vulneráveis. Com relação os fatores de risco pré-operatórios e intraoperatórios relacionados a readmissão, nosso estudo apontou a apneia do sono (OR: 1,117, IC 95%: 1,020 – 1,222, p=0,0165), arritmia cardíaca (OR: 1,040, IC 95%: 0,997 – 1,085, p=0,0712), uso de balão intra-aórtico (OR: 1,068, IC 95%: 1,024 – 1,113, p=0,0021), enquanto isso, a metanálise mais recente sobre a temática^15^ mostra correlação entre o desfecho e variáveis como sexo feminino, raça não caucasiana e assistência à saúde pela rede pública (*medicare* e *medicaid*), diabetes, hipertensão, infarto prévio do miocárdio, fibrilação atrial, acidente vascular encefálico, doença vascular periférica, disfunção renal, pulmonar, hepática, imunossupressão e obesidade.

Independentemente da diferença entre os resultados brasileiros e estrangeiros neste quesito, é importante ressaltar que a maioria das variáveis são características não modificáveis do paciente, desta forma, o corpo clínico hospitalar deve lançar mão do adequado planejamento de processos e condutas para a internação e prestação de cuidados a cada paciente, utilizando scores de risco, engajando o paciente e seus familiares sobre seus cuidados pós-cirúrgicos e realizando follow-up para investigação dos cuidados adequados e evolução clínica do doente.

O desenvolvimento de quadros infecciosos foram as razões mais expressivas de readmissão hospitalar, o que levanta a suspeita de que os tratamentos intra-hospitalares possam estar sendo insuficientes no que tange ao tratamento da infecção^16^ ou que os pacientes não estejam sendo orientados adequadamente com relação aos cuidados pós-operatórios. A relevância da infecção relacionada à readmissão hospitalar apresentada neste estudo vai de encontro com a literatura científica atual,^6-8,14^ enquadrando este achado em uma falha de coordenação após a alta hospitalar.

Nesta análise, observou-se que cada unidade adicional de hemoglobina intraoperatória (variável contínua do banco de dados) está associada a uma redução da probabilidade de readmissão hospitalar (OR 0,994, p= 0.000399). Ao mesmo passo que Trooboff et al.^6^ mostraram que o hematócrito <36% estão significativamente relacionado com o desfecho de readmissão (p=0.017).

Em nosso estudo, não foi avaliado o estado clínico dos pacientes além dos 30 dias de seguimento, entretanto, em 2021, Bianco et al.^5^ avaliaram os impactos a longo prazo da readmissão após a da cirurgia (n=14.538) e apontaram que a reinternação esteve significativamente associada à mortalidade tanto a curto (6 meses) quanto a longo seguimento (60 meses) além de ser um preditor independente para novas readmissões. Com relação a mortalidade neste presente estudo, o grupo de readmitidos apresentou 2 casos de óbitos durante a segunda internação (2/150, 1,33%), entretanto, sem correlação com o desfecho de readmissão pela análise estatística. Por outro lado, os não readmitidos não apresentaram óbitos na análise devido aos critérios de exclusão deste estudo.

No mundo atual, onde a assistência baseada em valor e a qualidade dos processos é cada vez mais valorizada,^17-19^ a *Affordable Care Act* (ACA)^20^ estabeleceu um programa de redução de readmissões hospitalares, visando reduzir pagamentos para centros de saúde com taxa de reinternação elevadas. A partir da mudança de paradigmas foi observado que as taxas de readmissão foram reduzidas, mostrando que este perfil de programa além de impactar no aumento da qualidade do serviço prestado aos pacientes, reduziu custos hospitalares.

O pagamento por desempenho vem ganhando cada vez mais destaque no mundo, o sistema baseia-se em ressarcir hospitais de acordo não apenas do seu volume cirúrgico, mas também avaliar aos resultados do hospital como complicações, óbitos e taxa de readmissão.^17-19,21^ Neste contexto, o Brasil, no primeiro semestre de 2022, lançou a portaria do QualiSus Cardio^18^ pelo Ministério da Saúde, se comprometendo a pagar até 45% a mais aos hospitais que tenham bom volume cirúrgico e bons resultados. Dentro deste cenário ter bons resultados passa a ser uma busca significativa pelas instituições que realizam cirurgias cardiovasculares. Análises como a avaliação da taxa de readmissão e os fatores que se associam ao desfecho tornam-se relevantes para a melhoria progressiva de performance pelas equipes de saúde, permitindo a otimização de processos futuros.

No que tange a linhas de processos modernos que lançam olhar sobre a melhoria nos atendimentos, os protocolos de rápida recuperação vêm progressivamente mostrando resultados positivos e promissores e apresentam potencial para reduzir ainda mais as taxas de readmissão nos centros de saúde. Em 2021, Chudgar et al.^22^ avaliaram o uso de um protocolo de desospitalização, que abordava o engajamento do paciente com relação aos cuidados com sua saúde e a intervenção cirúrgica a qual seria submetido, cuidados e gestão da doença de base e seguimento rigoroso após a alta hospitalar e concluíram que a linha de cuidados teve impacto significativo na redução de readmissões (14.1% versus 6.8%), gerando valor aos cuidados oferecidos aos pacientes.

A implementação deste tipo de linha de cuidados pode beneficiar o hospital de forma ampla,^23^ e tem potencial significativo para tal. A identificação das causas mais frequentes de readmissões hospitalares aponta que a resposta para a melhoria de resultados pode estar na melhoria da qualidade do atendimento institucional, beneficiando a saúde do paciente e deixando de trazer impacto negativo nos sistemas hospitalares pela utilização de recursos e material profissional para tais cuidados, além da questão orçamentária para o hospital.

### Limitações do estudo

Por se tratar de um estudo de coorte transversal no banco de dados multicêntrico REPLICCAR II, o desempenho de cada centro de saúde incluído na análise pode ter afetado a análise, devido a heterogeneidade de anos de experiência dos cirurgiões, do cardiologista clínico, materiais e outros. Os dados de acompanhamento sobre os casos de readmissão não estavam disponíveis além do exposto no presente artigo. Não fez parte do escopo do projeto atual, mas, é necessário avaliar como estão atualmente os pacientes que necessitaram readmissão após a alta de CRM, no que tange a qualidade de vida, estado clínico e evolução. Em uma próxima análise estes questionamentos devem ser levantados para a melhor compreensão do impacto da readmissão hospitalar para esta população de pacientes.

## Conclusão

Nesta análise de coorte transversal do banco de dados REPLICCAR II, 4,42% dos pacientes submetidos a CRM foram readmitidos até 30 dias após a alta hospitalar (N=150/3.392). O modelo múltiplo criado através das variáveis correlacionadas ao desfecho, se mostrou satisfatório para predizer readmissão hospitalar até 30 dias após a alta de cirurgia de CRM, evidenciada pela AUC de 0.70 da curva ROC, entretanto mais estudos devem ser conduzidos com o propósito de melhorar ainda mais a precisão preditiva do modelo.

Avanços em programas de qualidade e protocolos para cirurgias cardíacas enfatizam a necessidade de melhorar continuamente os resultados, visando reduzir as taxas de readmissão no estado de São Paulo, que embora inferiores às internacionais, têm grande potencial de aprimoramento.

## *Material suplementar

Para informação adicional, por favor, clique aqui.
