# Supplementary material for: Preditores de Readmissão Hospitalar até 30 Dias de CRM em Banco de Dados Multicêntrico: Estudo de Coorte Transversal
Source: Arq Bras Cardiol. 2024 Aug 21;121(9):e20230768. [Article in Portuguese] doi: 10.36660/abc.20230768 (PMC11495644; doi:10.36660/abc.20230768)
Supplement: Translation

# Translation: Predictors of 30-Day Hospital Readmission Following CABG in a Multicenter Database: A Cross-Sectional Study

**DOI:** 10.36660/abc.20230768i

**Authors:** Rene Augusto Gonçalves e Silva, Gabrielle Barbosa Borgomoni, Fabiane Letícia de Freitas, Adnaldo da Silveira Maia, Cleóstones Farias do Vale, Eva da Silva Pereira, Leonardy Guilherme Ibrahim Silvestre, Luís Roberto Palma Dallan, Luiz Augusto Lisboa, Luís Alberto Oliveira Dallan, Fabio Biscegli Jatene, Omar Asdrúbal Vilca Mejia, REPLICCAR Study Group

**Affiliations:** 1 Santarém PA Brazil Hospital João XXIII, Santarém, PA – Brazil; 2 São Paulo SP Brazil Instituto do Coração do Hospital das Clínicas da Faculdade de Medicina da Universidade de São Paulo, São Paulo, SP – Brazil; 3 São Paulo SP Brazil Instituto Dante Pazzanese de Cardiologia, São Paulo, SP – Brazil

**Keywords:** Hospitals, Patient Readmission, Myocardium

## Abstract

**Background:**

The analysis of indicators such as hospital readmission rates is crucial for improving the quality of services and management of hospital processes.

**Objectives:**

To identify the variables correlated with hospital readmission up to 30 days following coronary artery bypass grafting (CABG).

**Methods:**

Cross-sectional cohort study by REPLICCAR II database (N=3,392) from June 2017 to June 2019. Retrospectively, 150 patients were analyzed to identify factors associated with hospital readmission within 30 days post-CABG using univariate and multivariate logistic regression. Analysis was conducted using software R, with a significance level of 0.05 and 95% confidence intervals.

**Results:**

Out of 3,392 patients, 150 (4,42%0 were readmitted within 30 days post-discharge from CABG primarily due to infections (mediastinitis, surgical wounds, and sepsis) accounting for 52 cases (34.66%). Other causes included surgical complications (14/150, 9.33%) and pneumonia (13/150, 8.66%). The multivariate regression model identified an intercept (OR: 1.098, p<0.00001), sleep apnea (OR: 1.117, p=0.0165), cardiac arrhythmia (OR: 1.040, p=0.0712), and intra-aortic balloon pump use (OR: 1.068, p=0.0021) as predictors of the outcome, with an AUC of 0.70.

**Conclusion:**

4.42% of patients were readmitted post-CABG, mainly due to infections. Factors such as sleep apnea (OR: 1.117, p=0.0165), cardiac arrhythmia (OR: 1.040, p=0.0712), and intra-aortic balloon pump use (OR: 1.068, p=0.0021) were predictors of readmission, with moderate risk discrimination (AUC: 0.70).

## Introduction

The system and hospital processes performance evaluation through indicators help hospital management, contemplating not only the structural analysis of the institution but also the processes and results obtained over time, while the continuous quality interventions are applied in the health service.^1,2^ Initially, such indicators were focused on mortality and complications in patients, however, with knowledge advances, variables such as hospital readmission began to be included and evaluated by hospital managers.

The hospital readmission rate has currently played an important role in the quality management department of hospitals, as it is a performance indicator in the health care system, showing the "real-life analysis" related to the system quality, allowing the process monitoring that culminates in patient's readmission and definition of proactive strategies to improve results.^3^

The need for a newly operated patient to return to the hospital postpones his/her return to daily activities, causing difficulties in preserving health and general well-being from a psychological and physical point of view, exposing him/her again to the potentially contaminated hospital environment,^4^ increasing the probability of negative outcomes,^5^ especially in the new COVID-19 era,^4^ in addition to representing a financial impact on the hospital.

Readmissions are relatively common after coronary artery bypass graft (CABG) surgery, presenting heterogeneous data that oscillate between 8.3 and 21.1%,^6-8^ even with variations between institutions. Thus, there is a need to identify the reasons for this event, providing opportunities for proactive actions to reduce these rates shortly and better quality of care and assistance to patients undergoing CABG, in addition to greater availability of beds for new patients, consequently increasing health promotion for the population. Studies addressing this issue are still rare and scattered in Brazilian scientific literature, pointing to a significant variety of definitions and a gap in the knowledge of the medical community.

This study aims to identify predisposing factors for hospital readmission in patients undergoing CABG within 30 days after discharge. This is a cross-sectional cohort study, from the perspective of a retrospective analysis of the multicenter database of São Paulo state: the São Paulo Registry of Cardiovascular Surgery II (REPLICCAR II).

## Methods

### Study design

Cross-sectional study of REPLICCAR II,^9^ with a prospective, observational, and multicentric data collection from patients undergoing primary and isolated CABG between July 2017 and June 2019 in referral hospitals in São Paulo state.

The authors followed the Strengthening the Reporting of Observational Studies in Epidemiology (STROBE)10 guidelines.

For this analysis, we evaluated patients who needed to be readmitted to their health institutions within 30 days after hospital discharge following CABG, as well as the factors that predisposed them to hospital readmission after CABG surgeries.

The flowchart of the methodologies addressed is shown in Figure 1, while the characterization of the evaluated patients is represented in Supplementary File 1, comparing the profile and evolution of the population readmitted to the health institution and those who did not need to be readmitted.

**Figure 1:** Research flowchart. Source: own authorship.

### Data collection

We included all patients who underwent CABG during the selected period, over 18 years old, and with isolated and primary surgical indications (N=4,049). All data obtained were entered into the REDCap platform, in an area created for the REPLICCAR II project, by health professionals trained to perform this task. All variable definitions followed the criteria and definitions from the STS Adult Cardiac Surgery Database version 2.9.11 Quality audits were carried out periodically to verify data accuracy, integrity, and consistency.^12^

The variable morbidity was a composite outcome that included the following outcomes: cerebrovascular accident, acute kidney failure, prolonged intubation, deep sternal wound infection, and reoperation.

Exclusion criteria for this present analysis: Operative mortality (defined as deaths occurring after hospital discharge but within 30 days after the procedure, as well as deaths during the hospital stay in which the surgery was performed, even if 30 days after the procedure [N=133]) and missing data for "readmission" variable or life status until the 30^th^ day after the CABG discharge (N=524).

For this study, a cohort was selected among patients who needed to be readmitted to the health institution where they underwent CABG surgery (N=150), and based on their data, statistical regression analyses were performed to identify the causes related to readmission and the factors associated with the event. Data related to the 3.242 patients who were discharged non-30 days-readmission and were alive in the follow-up by the 30^th^ were compared with the readmitted group.

### Ethics and consent

This is a subanalysis of the REPLICCAR II project, approved by the Ethics Committee for Analysis of Research Projects (CAPPesq) of the *Hospital das Clínicas da Universidade de São Paulo*, with number 1575, under registration number SDC 4506/17/006. Informed consent was waived due to the methodology of the research design applied to the project.

### Statistical analysis

In the descriptive analysis, continuous variables were expressed as mean ± standard deviation or median and interquartile range, depending on the normality of the data, in terms of summary measures (mean, median, standard deviation, and quartiles), while categorical variables were expressed in terms of percentages. As continuous variables do not follow a normal distribution (Anderson-Darling test), for the comparison of two groups, the non-parametric Mann-Whitney and Brunner-Munzel tests were used for homogeneous and heterogeneous variables (Bartlett test), respectively. For categorical variables, the Fisher's Exact test or the Chi-Squared test was employed.

To find associations between explanatory variables and a binary outcome, the logistic regression model was utilized. For constructing more parsimonious multiple models (fewer variables), the stepwise backward algorithm was employed to select the variables that best explain the outcome. The AUC (Area Under the Curve) through the ROC Curve was used to evaluate the model's accuracy.

All analyses were conducted with the R software version 4.2.0. A significance level of 0.05 was adopted for the tests. Two-tailed hypotheses were considered. Additionally, the constructed confidence intervals are 95%.

## Results

A cross-sectional cohort was selected in the REPLICCAR II database, divided into two groups: 1) a Group of patients readmitted until thirty days after hospital discharge (N=150, 4.42% of the total sample of this analysis); and 2) Discharged patients who did not need to return to the hospital (N=3,242).

Supplementary table 1 shows the descriptive variables between the two groups analyzed. We observed that the group of patients who needed to be readmitted to the hospital until 30 days of discharge had a greater number of female patients (p<0.001), a predisposition to anemia in pre-, intra- and postoperative periods (p<0.001, p<0.001 and p=0.001), higher glycosylated hemoglobin levels (p=0.013 and need for intraoperative blood transfusion (p<0.001).

**Table 1:** Multivariate logistic regression model analysis for Hospital Readmission

Explanatory variable	Coefficient	Standard error Coefficient	OR	CI 95%	p-value
Intercept	0.093	0.015	1.098	1.065-1.131	<0.00001
Cardiac arrhythmia	0.039	0.022	1.040	0.997-1.085	0.071212
Sleep apnea	0.110	0.0046	1.117	1.021-1.222	0.016523
Lowest intraoperative hematocrit recorded during the intraoperative period	-0.006	0.002	0.994	0.991-0.997	0.000399
Intra-aortic balloon pump use	0.066	0.021	1.068	1.024-1.113	0.002103

*CI: confidence interval. Source: own authorship.*

We identified that, among the reasons that led patients to be readmitted to the institutions included in the study, infectious conditions (mediastinitis, surgical wound, and sepsis) were the most expressive (34.66%), as shown in Figure 2.

**Figure 2:** Causes of hospital readmission. Source: own authorship.

Regression analysis was performed using the outcome variable "Hospital readmission" in the REPLICCAR II database. Based on the significant variables of the univariate regression (Supplementary file 2), the multivariate model was constructed (Table 1). multivariate logistic regression model revealed variables with significant impact on the outcome of interest. The intercept was significant with an Odds Ratio (OR) of 1.098 (95% CI: 1.065 - 1.131, p<0.00001), indicating a baseline factor for the outcome when all other variables are held constant. Sleep apnea was associated with an 11.7% increased chance of the outcome (OR: 1.117, 95% CI: 1.020 - 1.222, p=0.0165). Cardiac arrhythmia showed an OR of 1.040 (95% CI: 0.997 - 1.085, p=0.0712), although this association did not reach statistical significance at the 0.05 level. The variable "lowest intraoperative hematocrit recorded during the intraoperative period" was associated with a slight decrease in outcome risk (OR: 0.994, 95% CI: 0.991 - 0.997, p=0.0004). Intra-aortic balloon pump use was associated with a 6.8% increase in the chance of the outcome (OR: 1.068, 95% CI: 1.024 - 1.113, p=0.0021).

To test the model performance to predict readmissions the forward and backward stepwise model was used. The ROC curve had an area under the curve (AUC) of 0.70, indicating a moderate discrimination capacity of the model (95% CI: 0.63 - 0.77). This AUC suggests that the model has a 70% probability of correctly discriminating between positive and negative outcomes (Figure 3).

**Figure 3:** ROC curve. Source: own authorship.

## Discussion

In the current analysis, it was observed that within the cohort from the REPLICCAR II database (N=3,392), there were 150 readmissions (N=4.42%). This rate is significantly lower than the observed in the current scientific literature. where readmission rates vary between 8.3 and 21.1%.^6-8^ We believe that this occurred because REPLICCAR II was premised on improving the quality of health services associated with the database. which brought positive advances in several indicators.^2,13^

In 2012. Li et al.14 evaluated 11.823 hospital discharges following CABG in California and identified a 30-day readmission rate of 13.2%, correlating the following risk factors: age, gender, family income, and zip code compatible with the most vulnerable regions. Regarding the pre and intraoperative risk factors related to readmission, our study pointed to sleep apnea (OR: 1.117, p=0.0165), cardiac arrhythmia (OR: 1.040, p=0.0712), and intra-aortic balloon pump use (OR: 1.068, p=0.0021), while the more recent meta-analysis on the subject15 shows a correlation between outcome and variables such as female gender. non-White ethnicity and lack of private health insurance (Medicare and Medicaid beneficiaries), diabetes, hypertension, previous myocardial infarction, atrial fibrillation, cerebrovascular accident, peripheral vascular disease, renal, pulmonary, and/or hepatic dysfunctions, immunosuppression, and obesity.

Regardless of the difference between Brazilian and foreign results in this matter, such variables are non-modifiable characteristics of the patient, therefore, the hospital clinical staff must use adequate planning of processes and conducts for the hospitalization and provision of care to each patient, using risk scores, engaging the patient and their families in their post-surgery care and performing follow-up to investigate the appropriate care and clinical evolution of the patient.

The development of infectious conditions was the most significant cause of hospital readmission, which raises the suspicion that in-hospital treatments may be insufficient regarding the treatment of infection16 or that patients are not receiving proper postoperative instructions. The relevance of infection related to hospital readmission presented in this study is in line with the current scientific literature,^6-8,14^ framing this finding in a lack of coordination after hospital discharge.

In this analysis, it was observed that each additional unit of hemoglobin (a continuous variable from the database), is associated with a decreased probability of hospital readmission (OR: 0.994, 95% CI: 0.991 - 0.997, p=0.0004). Trooboff et al.6 showed that hematocrit levels <36% are significantly related to the readmission outcome (p=0.017).

In our study, the clinical status of patients was not assessed beyond the 30-day follow-up period. However, in 2021 Bianco et al.^5^ assessed the long-term impacts of readmission (N=14.538) and pointed out that readmission was significantly associated with both short-term (6 months) and long-term (60 months) mortality, in addition to being an independent predictor of new readmissions. Regarding mortality in this study, the readmitted group recorded two cases of deaths, during the second hospitalization (2/150, 1.33%), however, these were not statistically correlated with the readmission outcome. On the other hand, the non-readmited group reported no deaths, due to the exclusion criteria of this study.

In a world where value-based care and quality processes are increasingly valued.^17-19^ the Affordable Care Act (ACA)20 established a program to reduce hospital readmissions, aiming to reduce payments to health centers with high readmission rates. With the change of paradigms. readmission rates were reduced. showing that this program profile. in addition to having an impact on increasing the quality of service provided to patients. reduced hospital costs.

Pay-for-performance is gaining increased prominence worldwide. The system is based on not only reimbursing hospitals according to their surgical volume but also evaluating hospital results such as complications. deaths and readmission rates.^17-19,21^ In this context. in the first half of 2022, Brazil launched the QualiSus Cardio18 ordinance by the Ministry of Health. committing to pay up to 45% more for hospitals that have a good surgical volume and good results. Within this scenario, having good results becomes a significant objective for institutions that perform cardiovascular surgeries. Analyzes such as evaluation of the readmission rate and factors associated with the outcome become relevant for the progressive performance improvement by the health teams. providing opportunities for the optimization of future processes.

Regarding types of modern processes that focus on improving care, rapid recovery protocols have progressively shown positive and promising results and have the potential to further reduce readmission rates in health centers. In 2021. Chudgar et al.^22^ evaluated the use of a discharge protocol that addressed patient engagement in health care and the surgical intervention to which they would be submitted, management of the underlying disease, and strict follow-up after hospital discharge. The authors concluded that the type of care had a significant impact on the reduction of readmissions (14.1% *vs*. 6.8%), adding value to the care offered to patients.

The implementation of this type of care can benefit the hospital in a broad way,^23^ and has significant potential. The identification of the most frequent causes of hospital readmissions points out that the answer to improving results may lie in improving the quality of institutional care, benefiting the patient's health, and no longer having a negative impact on hospital systems due to the use of resources and professional material for such care, in addition to the budgeting issue for the hospital.

### Study limitations

As this is a cross-sectional cohort study in the REPLICCAR II multicenter database, the performance of each health center included may have affected the analysis, due to the heterogeneity in years of experience of surgeons and clinical cardiologists, materials used, and others. Follow-up data on readmission cases were not available beyond what is set out in this work. It was not part of the scope of the current project; however, it is necessary to assess how patients who need to be readmitted after discharge following CABG surgery are currently doing in terms of quality of life, clinical status, and evolution. In a future analysis, these questions should be raised to better understand the impact of hospital readmission on this patient population.

## Conclusion

In this cross-sectional cohort analysis from the REPLICCAR II database, 4.42% of patients who underwent CABG were readmitted within 30 days post-discharge (N=150/3.392). The multiple models created through variables correlated with the outcome, proved satisfactory for predicting hospital readmission within 30 days post-CABG surgery, evidenced by the ROC curve's AUC of 0.70. However, further studies should be conducted to improve the model's predictive accuracy. The advancement of quality programs in cardiac surgeries with safety protocols shows the need and interest in the continuous improvement of results, which can positively affect the reduction of readmissions observed in São Paulo state, which are currently below the rates observed abroad. but with a great potential for improvement.

## *Supplemental Materials

For additional information, please click here.
